# Androgen receptor expression in metastatic adenocarcinoma in females favors a breast primary

**DOI:** 10.1186/1746-1596-1-34

**Published:** 2006-10-04

**Authors:** Vinod B Shidham, Richard A Komorowski, Jinobya K Machhi

**Affiliations:** 1 Department of Pathology; Medical College of Wisconsin, Milwaukee, WI, USA (JM was a cytopathology fellow during the study)

## Abstract

**Background:**

The differential diagnosis of metastatic mammary adenocarcinoma and adenocarcinomas from other primary sites can be challenging, particularly in tumors that are poorly differentiated and negative for Estrogen/Progesterone receptors (ER/PR). With progression of disease, Androgen receptors (AR) are preserved with higher frequency than ER/PR in metastatic mammary carcinoma. This study was undertaken to evaluate the diagnostic significance of AR expression in adenocarcinoma of breast and other morphologically similar adenocarcinomas.

**Design:**

Formalin-fixed paraffin-embedded tissue sections of 113 primary adenocarcinoma of various sites [breast (34, all females), lung (23, M- 6, F-17), colon (9, M-2, F-7), stomach (6, M-4, F-2), liver and bile duct (11, M-5, F-6), pancreas (7, M-2, F-5), ovary (10), endometrium (7), and cervix (6)] were immunostained with monoclonal antibody for AR. Except for well differentiated lobular carcinoma of breast (5) and bronchoalveolar carcinoma of lung (10), majority of the tumors were moderately to poorly differentiated. Tumors immunoreactive for ≥ 10% of nuclei were considered AR positive. However, AR immunoreactivity in the cytoplasm only was also recorded.

**Results:**

56% (19/34) mammary carcinoma and 20% (2/10) adenocarcinoma of ovary were positive for AR. Remaining 69 adenocarcinomas did not show nuclear immunoreactivity for AR in ≥ 10% nuclei; however, 52% (36/69) showed variable cytoplasmic immunoreactivity.

**Conclusion:**

Significant proportion of mammary carcinomas and some ovarian carcinomas express AR in the nuclei of more than 10% tumor cells. If metastatic tumor with unknown primary in a female is AR positive, breast and ovary are the most likely primary sites. Cytoplasmic immunoreactivity alone without nuclear immunoreactivity for AR was non-specific for this differential diagnosis.

## Background

Over the years with vast advances in the chemotherapeutic regimes specific for certain primary sites of tumors, the correct identification of the origin of tumor is of utmost importance for the determination of appropriate therapy and prognosis. In women, mammary carcinoma is a leading cause of death in several regions of the world [1]. It is an important differential in the evaluation of metastatic tumor especially in locations like axillary lymph nodes, lung, liver, and body fluids where it is one of the most common metastatic tumors in women [2].

The primary site of poorly differentiated metastatic adenocarcinoma may not always be discernible by morphology alone. Immunohistochemistry has proved to be a useful adjunct for this purpose. Commonly applied panels of immunomarkers for breast carcinoma including ER, PR, GCDFP-15 (Gross Cystic Disease Fluid Protein-15), and lactoferrin while very useful [3-6] may be inconclusive [7,8].

Previous studies have shown that AR is retained more often than ER/PR in metastatic mammary carcinoma [9,10], being the sole receptor in 25% of metastatic breast tumors in a report [9]. AR expression in primary breast cancer has been observed in 34–100% of cases in several reports [9,11-14]. The higher frequency of AR expression as compared to ER/PR makes it a promising addition to a panel of immunohistochemical markers for assessment of metastatic carcinomas. However, the status of AR by immunohistochemistry in other poorly differentiated carcinomas that may enter in the differential diagnosis for mammary carcinoma has not been evaluated previously. This study was undertaken to examine the expression of AR by immunohistochemistry in poorly differentiated primary breast carcinoma and adenocarcinomas from various other sites.

The tumors that are included in our study are primary adenocarcinomas from breast, lung, stomach, pancreas, liver (cholangiocarcinoma), colon, ovary, endometrium, and cervix.

## Materials and methods

A total of 113 cases were obtained after a computerized search from Anatomic Pathology files at Froedtert Hospital/Medical College of Wisconsin from 1996 to 2001. The neoplasms studied included the following: 34 mammary carcinomas (29 ductal, 5 lobular), 10 ovarian carcinomas, 7 uterine endometrial carcinomas, 6 uterine cervix adenocarcinomas, 11 liver and bile duct cholangiocarcinomas (M-5, F-6), 7 pancreatic adenocarcinomas (M-2, F-5), 6 gastric carcinomas (M-4, F-2), 9 colon carcinomas (M-2, F-7), and 23 adenocarcinomas of the lung (M-6, F-17) including 10 of the broncho-alveolar type. Majority of the tumors except for lobular carcinoma of the breast and broncho-alveolar carcinoma of the lung were of a moderately to poorly differentiated grade. All of these tumors were primary tumors of their respective organs. Out of the 56 adenocarcinomas (other than mammary carcinoma, ovarian carcinoma, uterine endometrial and cervical carcinomas of the female gender), 19 tumors belonged to males and 37 to females.

These specimens were fixed in 10% formalin and embedded in paraffin blocks. Sections of 3.5 micron in thickness were cut and mounted on DAKO silanized slides to accommodate alkaline epitope retrieval. They were then dried and depariffinized. The endogenous peroxidase activity was blocked using 50/50 methanol/H_2_O_2 _solution following which antigen retrieval is performed with DAKO pH 10.0 citrate buffer at 95°C for 20 minutes. After allowing it to cool down, non-specific binding was quashed with DAKO protein block. The tissue was then immunostained for 45 minutes with monoclonal antibodyAR441 (Dako, Carpinteria, CA) corresponding to amino acids 229–315 of the human androgen receptor at a dilution of 1:100. The signal was visualized with DAKO LSAB+. Then they were placed in DAKO DAB+ for 7 minutes. Lastly hematoxylin counter stain was performed using Harris Hematoxylin. Tumors were considered positive for AR if 10% or greater than 10% of nuclei were immunoreactive. Cytoplasmic reactivity alone with AR was interpreted as negative, but was recorded for analyzing the results. Formalin fixed paraffin-embedded tissue sections of prostate served as a positive control.

## Results

Of the 34 breast carcinoma cases, 29 were of infiltrating ductal type (IDC) and 5 of infiltrating lobular type (ILC). All of the IDC were assigned Bloom and Richardson grade 3. Nuclear immunoreactivity was observed in 56% (19/34) of the total breast cases. Out of the 29 cases of IDC, 14 were positive for AR. All 5 ILC were positive for AR. The staining pattern for AR was patchy in many of the tumors with proportion of tumor nuclei exhibiting immunopositivity for AR varying from 40–100%. The intensity also varied from weak to strong. In the ILC group, 4 cases displayed positive nuclear immunostaining with strong intensity, and in 1 case 75% of the nuclei stained positive with moderate to strong intensity. Simultaneous or isolated cytoplasmic staining with AR, ranging from weak to strong was noted in 62% (21/34) of these breast carcinomas.

The group of 10 ovarian tumors included 5 papillary serous adenocarcinomas, 3 endometriod carcinomas, 1 mixed papillary mucinous and serous adenocarcinoma, and 1 mixed endometriod – papillary serous adenocarcinoma. Immunoreactivity with AR was noted in 2 of these tumors, a papillary serous adenocarcinoma and the other mixed endometriod – papillary serous adenocarcinoma. Moderate to strong staining was noted in 80% and 95% of the tumor nuclei in these 2 tumors respectively. Nine out of the ten ovarian tumors displayed variable cytoplasmic immunoreactivity with AR.

Out of the 10 cases selected initially as colon adenocarcinoma, one tumor displayed AR immunoreactivity in scattered malignant glands but was negative in the poorly differentiated regions of the tumor. This tumor belonged to a male patient. Due to morphology unusual for colon carcinoma, further investigation with Prostatic specific antigen (PSA) immunomarker was performed to determine if this tumor had possible prostate origin. PSA immunoreactivity was observed in areas showing few well-differentiated malignant glands consistent with prostatic adenocarcinoma. The tumor was predominantly poorly differentiated and these poorly differentiated cells did not show AR immunoreactivity. Patient had a past history of prostatic adenocarcinoma. The rest of the colon adenocarcinoma cases showed non-reactivity of the nuclei with AR although variable cytoplasmic staining was noted in 7 of the 9 cases.

Amongst the 7 cases initially selected as gastric carcinoma, six did not display nuclear immunoreactivity for AR; however, one case belonging to a female patient showed moderate to strong positive nuclear staining with AR in 60% of the nuclei. Further immunohistochemical studies were performed which demonstrated immunoreactivity of the tumor cells for ER (20–30% nuclei with moderate intensity), PR (90% nuclei with strong intensity) and Cytokeratin 7. These tumor cells were negative for Cytokeratin 20, consistent with metastatic mammary carcinoma. Patient had a past history of mammary carcinoma. Cytoplasmic staining was noted in 3 out of the 6 gastric carcinoma cases.

Remaining 54 adenocarcinomas, including pancreatic adenocarcinomas (7), liver and bile duct cholangiocarcinomas (11), uterine endometrial carcinomas (7), uterine cervix adenocarcinomas (6), and pulmonary adenocarcinomas including broncho-alveolar type (23), did not show nuclear immunonegativity for AR. However 48% (26/54) of these tumors demonstrated variable cytoplasmic reactivity. Out of all 69 non-mammary/non-ovarian adenocarcinomas, 52% (36/69) showed variable cytoplasmic immunoreactivity.

## Discussion

Androgen receptors are nuclear proteins that are functionally critical to several organs and tissues [15]. They are expressed at variable levels in a number of tissues [16]. Intense staining has been reported in glandular epithelia of male accessory organs (including prostrate, seminal vesicles and epididymis), breast, and sebaceous and sweat glands of skin [17,18]. In the testes, sertoli cells, peritubular myoid cells and interstitial cells were immunoreactive with AR [17,18]. Stratified squamous epithelia of vagina and cervix showed selective immunostaining of the basal layer whereas in the preputial epithelia, the intensity of immunoreactivity decreased gradually with maturation [17].

AR expression has been noted in some benign and malignant tumors. As noted earlier, majority of breast carcinomas evaluated express AR as determined by IHC and biochemical studies [9,11-14]. In adenocarcinoma of the prostate, AR has been demonstrated by IHC with considerable heterogeneity in staining [18-21]. A significant number of ovarian carcinomas are also positive for AR [22,23]. Malignant cells in some endometrial adenocarcinomas were found to be immunoreactive [24]. AR has also been detected by IHC in 90% of salivary duct carcinomas [25,26]. Amongst the benign tumors, AR expression has been demonstrated in nasopharyngeal angiofibromas [27], hepatic adenomas [28], and meningiomas [29]. Recently, AR expression has been evaluated with reference to clinical and prognostic significance in estrogen receptor-negative breast cancers [30].

In our study there was a clear dominance of AR expression in mammary carcinoma compared to other adenocarcinomas. AR expression in 56% (19/34) of the mammary carcinomas is in concordance with previous studies. Whilst 48% (14/29) of the IDC's were immunoreactive for AR, ILC cases demonstrated consistent AR positivity at 100%. The higher frequency could be attributed to lower grade of the ILC's as compared to the poorly differentiated IDC's chosen in our study. It is noteworthy that 20% of ovarian carcinomas were positive with immunoreactivity for AR in more than 10% nuclei.

As specifically evaluated in this study, the nuclear immunostaining pattern is important during interpretation. Only nuclear immunostaining pattern was observed in mammary carcinoma and in a few cases of ovarian carcinoma. All other adenocarcinomas are negative for nuclear immunoreactivity. However, cytoplasmic immunoreactivity was not uncommon in many of these non-mammary/non-ovarian carcinomas.

Two cases (1 female and 1 male) showed positive immunoreactivity for AR in nuclei in the tumors initially selected in the non-mammary adenocarcinoma group. After further evaluation with additional immunomarkers and clinical correlation, these tumors were consistent with metastatic adenocarcinomas. The primary sites respectively were breast in a female and prostate in a male.

Addition of AR in the immunopanel for immunohistochemical evaluation of unknown primary in women is strongly recommended. Silanized slides are recommended to avoid floating and washing away of the tissue sections during alkaline epitope retrieval step. Depending on the clinical correlation, including the status of ovaries and breast, AR positivity favors primary from mammary carcinoma and sometimes ovarian carcinoma.

In summary, AR immunoreactivity in 10% or more nuclei is consistent with AR positive tumor, which is strongly suggestive of breast and in some cases ovarian primary. This study suggests that AR is a potentially useful immunomarker in evaluating metastatic adenocarcinoma of unknown primary in females. It is important to assess the true nuclear immunoreactivity in tumor cells. Cytoplasmic immunostaining alone is non-specific for differential diagnosis of these primary sites.

## Competing interests

The author(s) declare that they have no competing interests.

## Authors' contributions

JM designed, carried out entire study and prepared manuscript. VS conceived, designed and provided expertise in assisting in data collection and manuscript preparation as senior author and mentor. RK participated in the design and coordination of the study. All authors read and approved the final manuscript.

**Figure 1:**
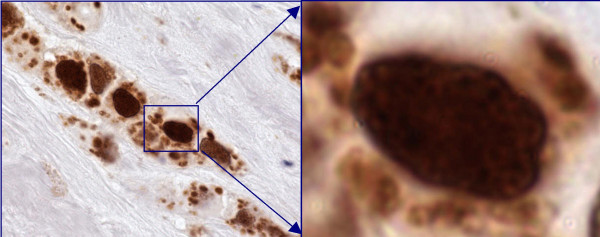
Mammary carcinoma- Positive for Androgen receptors (≥ 10% nuclei are immunoreactive, 100×).

**Figure 2:**
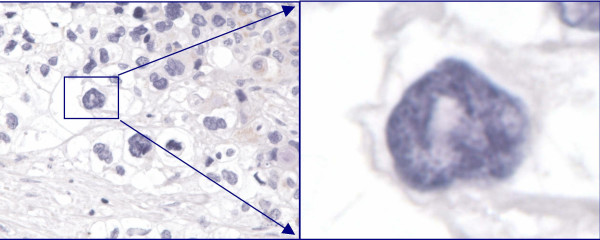
Pancreatic carcinoma- Negative for Androgen receptors (None to <10% nuclei are immunoreactive, 100×).

**Figure 3:**
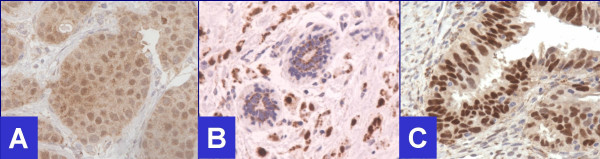
Positive for Androgen receptors. **A**. Infiltrating ductal carcinoma, **B. **Infiltrating lobular carcinoma, **C**. Ovarian carcinoma (40×).

**Figure 4:**
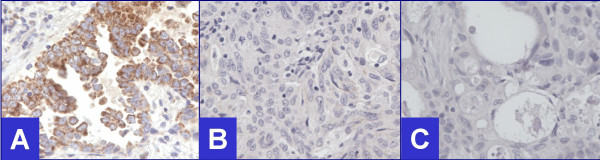
Negative for Androgen receptors (40×). **A. **Lung carcinoma (Nuclei- non-immunoreactive, Cytoplasm- immunoreactive), **B. **Endometrial carcinoma (Both, nuclei and cytoplasm- non-immunoreactive), **C. **Pancreatic carcinoma (Both, nuclei and cytoplasm- non-immunoreactive).

**Table 1:** AR positivity in different tumors

Tumor	Total (M+F)	AR Positive Nuclear or Nuclear-Cytoplasmic immunoreactivity %(n)	Cytoplasmic immunoreactivity
IDC	29 (All F)	48% (14/29)	55%(16/29)
ILC	5 (All F)	100% (5/5)	100% (5/5)
Ovary	10*	20% (2/10)	90% (9/10)
Endometrium	7	0% (0/7)	43%(3/7)
Cervix	6	0% (0/7)	17%(1/6)
Lung	23 (6 + 17)**	0% (0/23)	74%(17/23)
Colon	9 (2+7)***	0% (0/9)	78%(7/9)
Stomach	6 (4+2)****	0% (0/6)	50%(3/6)
Pancreas	7 (2+5)	0% (0/7)	43%(3/7)
Hepatobiliary	11 (5+6)	0% (0/11)	18%(2/11)

*Included 5 papillary serous adenocarcinomas, 3 endometrioid carcinomas, 1 mixed papillary mucinous and serous adenocarcinoma, and 1 mixed endometrioid and papillary serous adenocarcinoma.

** Included 13 poorly differentiated adenocarcinomas of lung and 10 bronchoalveolar carcinomas.

*** Initially 10 cases of colon adenocarcinoma were selected but one was excluded after concluding it to be metastatic prostrate adenocarcinoma to colon.

**** Initially 7 cases of gastric carcinoma were selected but one was later excluded following conclusion of it being metastatic mammary carcinoma to stomach.
